# Prevalence and determinants of uncontrolled asthma among secondary school-going adolescents in Kampala City, Uganda: findings from a cross-sectional study

**DOI:** 10.1186/s12887-025-06292-2

**Published:** 2025-11-12

**Authors:** James Davis Katumba, Bruce Kirenga, Florence Nanteza, Ruth Mpirirwe, Alex Mwesigwa, Anthony Muwagga Mugagga, Joan N. Kalyango, Charles Karamagi, Rebecca Nantanda

**Affiliations:** 1 Clinical Epidemiology Unit, College of Health Sciences, Makerere University, P O Box 7072, Kampala, Uganda; 2 Lung Institute, College of Health Sciences, Makerere University, Kampala, Uganda; 3 Department of Internal Medicine, College of Health Sciences, Makerere University, Kampala, Uganda; 4 Uganda Cancer Institute, Kampala, Uganda; 5 Department of Microbiology and Immunology, School of Medicine, Kabale University, Kabale, Uganda; 6 College of Education and External Studies, Makerere University, Kampala, Uganda; 7 Department of Pharmacy, School of Health Sciences, Makerere University, Kampala, Uganda; 8 Health Tutors’ College Mulago, Kampala, Uganda

**Keywords:** Uncontrolled asthma, Adolescents, Kampala

## Abstract

**Background:**

The Uganda Ministry of Health Sector Performance Report 2022 highlights a salient increase in non-communicable, genetic and environmentally engineered diseases such as asthma among Ugandans, mostly the children. Much of the effort towards controlling asthma is geared towards early and middle childhood when asthma is more frequent. Adolescents in school, face a high risk of poor asthma control due to limited health care access, inadequate school support, and low adherence to treatment. However, uncontrolled asthma among adolescents aged 12–19 years in Kampala is under studied. This study investigated the prevalence and determinants of uncontrolled asthma among adolescents in selected secondary schools in Kampala City, Uganda.

**Methods:**

This cross-sectional study involved 263 secondary school adolescents who had a documented history of asthma in Kampala city, Uganda. The participants provided written informed consent or assent with parental/legal guardian consent. Multistage sampling was done to select the participating secondary schools and adolescents with asthma. Data were collected using self-administered questionnaires based on the International Study of Asthma and Allergies in Childhood (ISAAC) and the Asthma Control Test (ACT). Asthma control was categorized as well controlled (ACT ≥ 20) or not well controlled (“Uncontrolled”, ACT ≤ 19). Descriptive statistics and bivariate analyses were performed, followed by multivariate mixed-effects logistic regression models to account for clustering of participants within schools and identify determinants of uncontrolled asthma.

**Results:**

The study found poor asthma control in 81.8% of the enrolled adolescents. The environmental determinants influenced asthma control; adolescents exposed to moulds were four times more likely to have uncontrolled asthma (AOR = 3.84, 95% CI: 1.02, 14.46; *p* = 0.047), and reliance on public transport significantly increased the odds of having uncontrolled asthma (AOR = 5.75, 95% CI: 1.38, 24.05; *p* = 0.016).

**Conclusion:**

Self-reported uncontrolled asthma was observed among secondary school adolescents with a documented history of asthma in Kampala. Environmental factors, such as reliance on public transport and household mould exposure, significantly contributed to poor asthma control. To address these, efforts should focus on reducing exposure through improved home ventilation and mould control, promoting safer and less polluted commuting options for students, and enhancing school-based asthma management. We recommend regular asthma screening, teacher training, and better access to care in schools to enhance its early detection and control.

## Introduction

Asthma, which affects approximately 262.41 million people globally [69], is a heterogeneous disease usually characterized by chronic airway inflammation. Asthma is defined by a history of respiratory symptoms such as wheeze, shortness of breath, chest tightness, and cough that vary over time and in intensity, together with variable expiratory airflow limitation [21]. The goal of asthma therapy is to achieve good symptom control and minimize risks including mortality, future exacerbations, persistent fixed airflow limitation, and treatment side effects [20]. Despite the availability of effective treatments such as inhaled corticosteroids (ICSs) and short- and long-acting β2-agonists, asthma in many children and adolescents remains uncontrolled [23, 28].

Globally, uncontrolled asthma affects 45–46% of asthma patients [41]. In developed countries, asthma in 40–70% of asthma patients is insufficiently controlled [57]. However, the prevalence of uncontrolled asthma in East Africa is unknown [31]. In Uganda, 67% of asthma patients seeking care from hospitals have uncontrolled asthma [61]. In 2019, 45% of Ugandan urban school children aged 5–17 years with asthma had uncontrolled asthma [43]. However, the current prevalence of uncontrolled asthma among Ugandan adolescents aged 12–19 years is not clear. The prevalence of uncontrolled asthma is increasing in low- and middle-income countries [18], with urbanization and industrialization contributing to the rise due to environmental pollution, sedentary lifestyles, obesity and unhealthy diets [70]. The Uganda National Population Census in 2024 revealed a sharp increase in industrialization and urbanization, along with their diverse effects on Kampala and its immediate residents [66]. Reports such as the Uganda Ministry of Health Annual Health Sector Performance Report 2022, allude to the fact that there is salient increase in non-communicable, genetic and environmentally engineered diseases such as asthma among Ugandans mostly the children [67].

Literature highlights several determinants of uncontrolled asthma among adolescents. Medication-related determinants of poor asthma control include errors in medication use, such as not prescribing inhaled corticosteroids (ICS), poor treatment adherence, absence of an action plan, and inadequate inhaler technique or disease education [13]. Biological or genetic determinants of uncontrolled asthma are indicated by a parental or sibling history of the condition [15]. Environmental determinants of asthma control include exposure to triggers such as smoking, cigarette smoke, allergens (if sensitized), and continued exposure to infectious agents (especially viruses) and allergens [11]. Comorbidities such as obesity, chronic rhinosinusitis, and gastroesophageal reflux disease also contribute to poor asthma control [68]. Family-related determinants of asthma control include exposure to parental stress, poor family functioning, physical inactivity, and unhealthy diets [35]. Additionally, sociodemographic determinants like a lower educational status, a child’s early residence, and contact with farm animals further increase the likelihood of uncontrolled asthma [37].Uncontrolled asthma imposes a significant social and economic burden, leading to absenteeism, physical activity limitations, and high healthcare resource utilization [24]. In adolescents, it is associated with increased disability, reduced productivity and health-related quality of life [9].

Approximately half of adolescents with asthma struggle with medication adherence, making uncontrolled asthma more common in this age group [28]. Compared to younger children, adolescents with asthma face unique challenges, including behavioral and emotional difficulties that contribute to poor management and non-adherence [27, 55].

The school environment plays a critical role in asthma management [22]. Teachers and school staff are often unprepared to handle asthma emergencies, and the presence of triggers such as dust, fumes, and allergens in schools can affect asthma symptom control [22]. Delayed or inappropriate responses to asthma emergencies reportedly lead to poor asthma control in schools [1]. Additionally, in low- and middle-income countries, chronic non-communicable diseases such as asthma are often sidelined by competing conditions such as infectious diseases and malnutrition [2].

The goal of improving asthma management requires identifying determinants of poor asthma control. Despite growing evidence of rising asthma prevalence linked to urbanization and environmental changes in Uganda, there remains a critical gap in knowledge regarding the current prevalence and determinants of uncontrolled asthma specifically among secondary school-going adolescents aged 12–19 years in Kampala City. Establishing context-specific determinants can help guide the design of interventions and improve patient quality of life for adolescents with asthma. In Uganda, studies on asthma in children have traditionally focused on issues such as prevalence, Human Immunodeficiency Virus (HIV) and asthma interactions, control of asthma and allergies among children [32, 33, 40, 42, 43, 47, 61]. However, more data are needed on asthma control, particularly among school-going adolescents, to develop ethnic and social context-specific interventions and management strategies. Therefore, this study aimed to close this knowledge gap by establishing the prevalence and determinants of uncontrolled asthma among adolescents in selected secondary schools in Kampala city, Uganda.

## Methods

### Study design and setting

This study adopted a cross-sectional research design with quantitative methods of data collection conducted between February to November 2024 in selected public and private, boarding and day, mixed and single secondary schools in Kampala city, Uganda. Kampala city experiences a tropical climate characterized by two distinct rainy seasons (March–May and September–November), consistently high humidity, and elevated levels of air pollution. The annual mean concentration of fine particulate matter (PM_2.5_, particles with an aerodynamic diameter ≤ 2.5 µm) is estimated at 29.1 µg/m^3^ [26], nearly three times higher than the World Health Organization (WHO) recommended guideline value [72]. The main sources of this pollution include industrial emissions, motor vehicle exhaust, biomass fuel combustion, and open waste burning [30, 51]. Kampala city hosts 295 secondary schools with 106,905 students aged 13–18 years [29, 65], many of whom reside in densely populated urban slums characterized by substandard housing, poor sanitation, and limited access to healthcare services [17]. The day students travel to and from school and spend eight hours daily in the school environment while boarding students reside in school dormitories or hostels near the schools. Each school is required to have a school nurse who provides first aid and attends to the sick children.

### Study population, eligibility criteria, sample size calculation and sampling procedure

The study targeted students in Senior One to Senior Six (S. 1–S. 6), which in Uganda corresponds to lower secondary (S. 1–S. 4; ages 12–16 years) and upper secondary (S. 5–S. 6; ages 17–19 years). From this population, 263 adolescents aged 12–19 years enrolled in secondary schools in Kampala City, Uganda, participated in the study. Adolescents with a documented history of asthma who were present at school during the study were eligible. Students who were absent on the days of the study and those who were too sick to provide responses to the questionnaires were excluded.

### Study sample

We estimated sample size for the two objectives: (1) establishing the prevalence and (2) identifying determinants of uncontrolled asthma among adolescents in selected secondary schools in Kampala City, Uganda. For the first objective, we used the modified Kish Leslie formula [34], incorporating both a design effect and a nonresponse adjustment.

Using a previously reported prevalence of uncontrolled asthma of 45% (*p* = 0.45) among Ugandan children [43], with a 95% confidence level (Z = 1.96), a precision of 5% (d = 0.05), a design effect of 2, and a 10% nonresponse rate, the calculated sample size was 845.

However, a finite population correction was applied based on a preliminary survey that identified 375 adolescents with asthma, yielding a final sample size of approximately 260 participants.

For the second objective—identifying determinants of uncontrolled asthma among adolescents in selected secondary schools in Kampala City—we used Rosner’s formula [59] for comparing two proportions, incorporating a design effect and a nonresponse adjustment. A Swedish population-based study reported a higher prevalence of uncontrolled asthma among girls (38.3%) than boys (25.2%) [63]; we used these values as p₁ = 0.383 and p₂ = 0.252, with group proportions q₁ = 0.52 and q₂ = 0.48. With a 95% confidence level (Zα = 1.96), 80% power (Zβ = 0.84), a design effect of 2, and a 10% nonresponse rate, the required sample size was 880 participants. Due to resource constraints, we recruited 263 participants, which was sufficient to meet the first objective and allowed analysis of determinants of uncontrolled asthma.

### Sampling procedure

We used multistage sampling in this study. We obtained a list of 99 schools in Kampala city from the Ministry of Education’s Uganda Certificate of Education (UCE) selection database, and eliminated 13 schools that had incomplete records [39]. We classified eligible schools by level (ordinary, advanced, or both), gender composition (mixed, girls, or boys), and section type (day, boarding, or both). We used a sampling fraction of 20% in primary sampling equivalent to 17 schools following the recommendations of Turner, Yansaneh, and Jambwa [64]. We used random numbers generated with Microsoft Excel’s “rand” function to select the schools from the seven categories. In secondary sampling, the individual participants were consecutively sampled until the required sample size was achieved for each school category. The number of participants selected from each school was calculated basing on their recorded number of candidates presented between 2011–2016 in the Uganda Certificate of Education and or Uganda Advanced Certificate of Education open dataset [45]. We selected one girls' boarding non-universal school with 18 participants (6.84%), two private girls' boarding non-universal schools with 37 participants (14.07%), two mixed-gender boarding non-universal schools with 32 participants (12.17%), five mixed-gender day non-universal schools with 70 participants (26.62%), three mixed-gender day universal schools with 66 participants (25.1%), one private mixed-gender day non-universal school with six participants (2.28%), and three mixed-gender day-boarding non-universal schools with 34 participants (12.93%). An independent statistician from the School of Statistics and Planning (SSP), College of Business and Management Sciences (CoBAMS), Makerere University, who had no prior knowledge of the schools or students involved, led this process.

### Study variables

The dependent variable in this study was asthma control, measured using the standardized Asthma Control Test (ACT) and categorized as either well-controlled (ACT score ≥ 20) or uncontrolled (ACT score ≤ 19). We grouped independent variables into sociodemographic, environmental, clinical, and behavioral domains. Sociodemographic data included age, sex, class; school section (boarding/day), area of residence (urban/rural), years lived in Kampala, birth in Kampala, parental marital status, and guardian education levels. Environmental factors included household exposure to mould and rats, cooking fuel type (electricity, gas, or open fire), and means of transport to school, serving as proxies for environmental and socioeconomic context. Clinical variables captured chronic illness status and type, age at asthma diagnosis, use of a preventer inhaler, and type of healthcare access (formal, informal, or none). Asthma symptom history encompassed wheezing, sleep and speech disturbances, exercise-induced wheezing, cough without infection and annual asthma attacks. Family history included presence of asthma among maternal, paternal, or sibling relatives. Behavioral and lifestyle factors covered dietary habits (burgers, tomatoes, sweet red wine), physical activity frequency, paracetamol use, and screen time (television hours during holidays). We assessed access to care using distance and time to the nearest health facility from home and school. Finally, anthropometric data included weight in kilograms (kgs) and height in metres (m) to calculate the Body Mass Index (BMI), classified using WHO standards into underweight, healthy weight, overweight, or obese [73].

### Data collection tools

Data was collected using the adapted versions of the International Study of Asthma and Allergies in Childhood (ISAAC) core and environmental questionnaires [5]. The ISAAC questionnaires have been widely used to assess asthma prevalence and risk factors among adolescents in African countries, including Uganda, Kenya and South Africa [2, 7, 43]. Asthma control was assessed via the Asthma Control Test (ACT), which assigns a numerical score to evaluate whether asthma symptoms are well-controlled [49]. The ACT has been validated and or used in low income countries including Uganda [43, 56, 61], Zimbabwe [50], South Africa [19], Nigeria [53] and Tanzania [62] with a documented sensitivity and specificity of 71.0% in adolescents [48].

### Data collection procedure

We obtained permission from the Head teachers of the selected schools to conduct the study and to collaborate with the school nurse(s) or health coordinators. We explained the purpose of the study to the students during a general assembly. We invited students aged 12–19 years with a documented history of asthma in the past year, as identified by school nurses basing on the students’ termly medical form or other medical reports, to participate. Eligible students who consented/assented and whose parents consented were consecutively sampled and subsequently completed self-administered questionnaires under the researchers’ guidance in a designated hall, tree shade or classroom at their schools.

### Data management and analysis

We reviewed the questionnaires for completeness, coded the data, and entered them into KoboToolbox. The data were then exported to Excel and subsequently imported into STATA version 14 for analysis [60]. Asthma Control Test (ACT) scores were classified as not well controlled/uncontrolled asthma (ACT ≤ 19), and well-controlled asthma (ACT ≥ 20) [49]. We estimated the prevalence of uncontrolled asthma, and descriptive summaries of the ISAAC questionnaire data were presented. Univariate analysis was used to determine proportions for categorical variables, means (± SD) for normally distributed continuous variables, and medians with interquartile ranges (IQRs) for asymmetrically distributed variables. To identify determinants of uncontrolled asthma, we used multivariate mixed-effects logistic regression, with models fitted using the binomial family and logit link function where odds ratios (ORs) and 95% confidence intervals (CIs) were calculated. School category and school were included in the model as random intercepts. We retained variables with *p* ≤ 0.2 from the bivariate analysis for further evaluation. Variables with *p* < 0.05 in the bivariate model, along with those supported by biological plausibility and previous literature, were included in the base multivariable model. We formulated and tested each two-way interaction term by adding it individually to the model and assessed its significance (*p* < 0.05) and impact on model fit. We then added the remaining variables (with 0.05 < *p* ≤ 0.2) one at a time to assess confounding, defined as a ≥ 10% change in the odds ratio of any primary predictor. We considered adjusted ORs with *p* < 0.05 to be statistically significant. We controlled for differences in school environments during the study by sampling across school categories and adjusted for clustering and sampling fractions in the analysis.

## Results

We enrolled 263 participants. The mean age was 16.2 (SD ± 1.7) years. They had lived in the city for a median of 15 years (IQR: 12–17). Females made up 78.7% of the sample. Most participants resided in urban areas (91.2%), and 68.8% attended boarding schools. The class distribution revealed that S. 3 was the most represented (29.7%), followed by S. 2 (22.8%) and S. 1 (20.2%). Transport to school was carried out mainly by private cars (54.4%), and 75.4% were born in Kampala. With respect to parental education, 77.8% of fathers and 68.7% of mothers had obtained tertiary education. The predominant family structure was married and living together (64.7%) Table 1.

**Table 1:** Sociodemographic characteristics of the adolescents who completed the asthma control test

Variable	Category	Response
Age (263)	Mean(± SD) [95% CI]	16.15 (± 1.75) [15.94–16.36]
Years lived in the City (226)	Median (IQR)	15 (IQR: 12–17)
Sex (263)	Female	207(78.7%)
	Male	56(21.3%)
Class (263)	S. 1	53(20.2%)
	S. 2	60(22.8%)
	S. 3	78(29.7%)
	S. 4	21(8.0%)
	S. 5	26(9.9%)
	S. 6	25(9.5%)
Section (263)	Boarding	181(68.8%)
	Day	82(31.2%)
Area of residence (261)	Rural	23(8.8%)
	Urban	238(91.2%)
Means of transport to school (261)	Foot	51(19.5%)
	Motor cycle bicycle	23(8.8%)
	Other	7(2.7%)
	Private car	142(54.4%)
	Public taxi bus	38(14.6%)
Marital status of parents (258)	Married living together	167(64.7%)
	Separated divorced	61(23.6%)
	Single	21(8.1%)
	Widowed	9(3.5%)
Male guardian/Father’s education (261)	None	2(0.8%)
	Primary school	8(3.1%)
	Secondary school	48(18.4%)
	Tertiary	203(77.8%)
Female guardian/Mother’s education (262)	None	6(2.3%)
	Primary school	20(7.6%)
	Secondary school	56(21.4%)
	Tertiary	180(68.7%)
Born in Kampala (260)	Yes	196(75.4%)

### Clinical characteristics

Asthma onset was at a median of nine years (IQR: 5–13). The median distance to the nearest health facility from home was two km (IQR: 1–3), whereas the median distance from school one km (IQR: 0.5–2). The median time to reach the facility from home was 20 min (IQR: 10–32.5), whereas that from school was 10 min (IQR: 5–30). A majority (67.2%) reported a chronic illness, with allergies (40.3%) being most common. In the past year, 44.4% of the participants experienced sleep disturbances, and 86.1% reported speech disturbances due to asthma. The level of physical activity was low, with only 16.0% exercising three or more times weekly. The body mass index (BMI) indicated that the majority (69.1%) had a healthy weight, while 17.0% were overweight, 8.1% underweight, and 5.8% obese Table 2.

**Table 2:** Clinical characteristics of the adolescents who completed the asthma control test

Variable	Category	Response
Age when asthma was first detected (206)	Mean(± SD) [95% CI]	8.65(± 4.82) [7.99–9.31]
Distance to the nearest health facility from home in Kms (193)	Median (IQR)	2 (IQR: 1–3)
Distance to the nearest health facility from school in Kms (239)	Median (IQR)	1 (IQR: 0.5–2)
Time to health facility from School in minutes (242)	Median (IQR)	10 (IQR: 5–30)
Time to Health facility from home in minutes (224)	Median (IQR)	20 (IQR: 10.0–32.5)
Number of attacks in the previous year (141)	Median (IQR)	5 (IQR: 3–10)
Television hours during holiday (216)	Median (IQR)	8 (IQR: 4–14)
Chronic illness (262)	Yes	176(67.2%)
Chronic illness Specified (154)	Allergies	62(40.3%)
	Stress depression and anxiety	22(14.3%)
	Gastroesophageal reflux disease	13(8.4%)
	Obstructive sleep apnea	1(0.7%)
	Other	22(14.3%)
	Rhinitis and or sinusitis	34(22.1%)
Having a preventer inhaler	Yes	205(79.5%)
Seeks health care from (259)	Formal care	239(92.3%)
	Informal care	11(4.3%)
	No care	9(3.5%)
Wheezing in the previous 12 months (263)	Yes	260(98.9%)
Sleep disturbance in the last 12 months (257)	Never woken with wheezing	38(14.8%)
	Less than one night per week	114(44.4%)
	One or more nights per week	105(40.9%)
Speech disturbance in the past 12 months (259)	Yes	223(86.1%)
Wheezing after exercise (261)	Yes	248(95.0%)
Cough without chest infection (263)	Yes	246(93.5%)
Family members with asthma (261)	Yes	192(73.6%)
Family members with asthma (192)	Maternal	72(37.5%)
	Paternal	75(39.1%)
	Siblings	45(23.4%)
Used paracetamol in the previous month (260)	Never	13(5.0%)
	At least once a year	60(23.1%)
	At least once per month	187(71.9%)
Cooking fuel (261)	Electricity	45(17.2%)
	Gas	75(28.7%)
	Open fires	141(54.0%)
Physical activity (262)	Never or occasionally	79(30.1%)
	Once or twice per week	141(53.8%)
	Three or more times a week	42(16.0%)
BMI (259)	Underweight (less than 18.5)	21(8.1%)
	Healthy weight (18.5—24.9)	179(69.1%)
	Overweight (25.0—29.9)	44(17.0%)
	Obesity (30.0 and above)	15(5.8%)
Raw Tomatoes (261)	Never or Occasionally	111 (42.5%)
	Once or Twice per Week	97 (37.2%)
	Three or More Times per Week	53 (20.3%)
Burgers (261)	Never or Occasionally	134 (51.3%)
	Once or Twice per Week	82 (31.4%)
	Three or More Times per Week	45 (17.2%)
Sweet Red Wine (260)	Never or Occasionally	236 (90.8%)
	Once or Twice per Week	19 (7.3%)
	Three or More Times per Week	5 (1.9%)
Moulds presence in a home (261)	Yes	57 (21.8%)
Rats presence in a home (261)	Yes	139 (53.3%)

### Asthma control

We categorized the total asthma control test scores for the 263 participants into two levels, 81.8% (215) [95% CI: 76.6–86.0%] were classified as not well controlled (≤ 19), whereas 18.3% (48) [95% CI: 13.8–23.5%] had well-controlled asthma (≥ 20). Of the adolescents studied, 175 females (84.5%) and 40 males (71.4%) had uncontrolled asthma, while 32 females (15.5%) and 16 males (28.6%) had controlled asthma.

Years lived in Kampala city, sex, section, area of residence, means of transport to school, being born in Kampala, age when asthma was detected, time to health facility from school (in minutes), time to health facility from home (in minutes), use of paracetamol in the previous one month, cooking fuel, consumption of burgers, presence of moulds in the home, and presence of rats in the home were variables with *p*-values less than or equal to 0.2 in the bivariate mixed-effects logistic regression. In the multivariate mixed-effects logistic regression, exposure to moulds and means of transport to school were statistically significant variables. Environmental determinants such as the means of transport to school influence asthma control, as students who rely on public transport have significantly increased odds of having uncontrolled asthma (AOR = 5.75, 95% CI: 1.38, 24.05; *p* = 0.016) Table 3. Compared with other respondents, those with moulds present in their homes were four times more likely to have uncontrolled asthma (AOR = 3.84, 95% CI: 1.02, 14.46; *p* = 0.047).

**Table 3:** Multivariate mixed-effects logistic regression model for the determinants of uncontrolled asthma among school going adolescents in Kampala City

**Variable**		**Uncontrolled**	**Controlled**	Unadjusted Odds Ratio (UOR* [95% CI]**)	**p > z**	Adjusted Odds Ratio (AOR*** [95% CI])	**p > z**
**Means of transport**	Private car (142)	116 (81.7%)	26 (18.3%)	**Reference**			
	Foot (51)	47 (92.2%)	4 (7.84%)	0.37 [0.11, 1.24]	0.106	0.20 [0.01, 3.18]	0.252
	Motor-cycle/Bicycle (23)	21 (91.3%)	2 (8.7%)	0.42 [0.09, 1.95]	0.267	0.58 [0.05, 7.00]	0.665
	Other (7)	6 (85.7%)	1 (14.3%)	0.64 [0.07, 5.77]	0.694	11.21 [0.71, 175.97]	0.085
	Public taxi/Bus (38)	25 (65.8%)	13 (34.2%)	**2.69 [1.13, 6.38]**	**0.025**	**5.75 [1.38, 24.05]**	**0.016**
**Moulds**	No (204)	172 (84.3%)	32 (15.7%)	**Reference**			
	Yes (57)	41 (71.9%)	16 (28.1%)	**2.22 [1.08, 4.53]**	**0.029**	**3.84 [1.02, 14.46]**	**0.047**
**Rats**	No (122)	106 (86.9%)	16 (13.1%)	**Reference**			
	Yes (139)	107 (77.0%)	32 (23.0%)	**2.12 [1.07, 4.21]**	**0.031**	2.44 [0.69, 8.69]	0.168
**Section**	Boarding (181)	143 (79.0%)	38 (21.0%)	**Reference**			
	Day (82)	72 (87.8%)	10 (12.2%)	0.52 [0.23, 1.17]	0.114	0.62 [0.12, 3.28]	0.576
**Born in Kampala**	No (64)	46 (71.9%)	18 (28.1%)	**Reference**			
	Yes (196)	167 (85.2%)	29 (14.8%)	**0.45 [0.23, 0.90]**	**0.024**	0.64 [0.10, 4.17]	0.639
**Burgers**	Never or Occasionally (134)	107 (79.9%)	27 (20.2%)	**Reference**			
	Once or Twice/Week (82)	64 (78.1%)	18 (22.0%)	1.19 [0.59, 2.39]	0.628	2.87 [0.80, 10.35]	0.106
	Three + Times/Week (45)	42 (93.3%)	3 (6.7%)	0.31 [0.09, 1.09]	0.068	0.75 [0.12, 4.83]	0.764
**Time to hospital from school**	Mean ± SD (n)	17.89 ± 18.69 (*n* = 199)	13.47 ± 14.39 (*n* = 43)	0.98 [0.96, 1.01]	0.144	0.98 [0.95, 1.02]	0.325
**Time to hospital from home**	Mean ± SD (n)	28.52 ± 23.91 (*n* = 184)	22.70 ± 19.05 (*n* = 40)	0.99 [0.97, 1.01]	0.210	1.01 [0.98, 1.04]	0.482
**Age when asthma was detected**	Mean ± SD (n)	8.86 ± 4.79 (*n* = 177)	7.38 ± 4.88 (*n* = 29)	0.94 [0.86, 1.02]	0.122	0.97 [0.86, 1.06]	0.584
**Years in Kampala**	Years in Kampala	13.78 ± 5.6 (*n* = 188)	11.97 ± 5.70 (*n* = 38)	0.94 [0.89, 1.00]	0.069	0.98 [0.85, 1.14]	0.840

Adjusted Odds Ratios (AORs) were obtained from a single multivariate mixed-effects logistic regression model. Confounding was observed for moulds (time to hospital from home, age when asthma was detected, rats) and for means of transport (years in Kampala, time to hospital from home and school, age when asthma was detected, burgers consumption and section for public taxi)

Statistically significant *p*-values (*p* < 0.05) are in bold

^*^Unadjusted Odds Ratio (UOR)

^**^95% Confidence Interval (95% CI)

^***^Adjusted Odds Ratio (AOR)

## Discussion

Asthma control among adolescents varies significantly worldwide due to variations in environmental triggers, genetics, chronic conditions, use of medicines, sociodemographic determinants, cultural influences, healthcare access, and lifestyle behaviors. This study investigated the prevalence and determinants of uncontrolled asthma among adolescents in selected secondary schools in Kampala city, Uganda. The study revealed that 81.8% [95% CI: 77.1–86.4%] (215 out of 263 participants) of adolescents with a documented history of asthma were classified as not well controlled or uncontrolled (Asthma Control Test score ≤ 19). This high prevalence of poor asthma control suggests a substantial burden of uncontrolled asthma among adolescents, emphasizing the need for targeted interventions. The high prevalence of uncontrolled asthma could also have been due to the sampling procedure where only adolescents with documented history of asthma by the school nurse were included in the study. Our findings are similar to those of Rishi et al. [58] who found asthma not controlled in 80% of the asthmatic respondents in Saudi Arabia despite it being a high income country. Our finding contrasts with findings from various regions. In London, 49.6% of secondary school children with a median age of 13 years had uncontrolled asthma [25], whereas in Jordan, 100% of high school students (mean age 14.5 years) were reported as having uncontrolled asthma [3] and in Portugal 38% in 12–17 years’ adolescent students from a Portuguese primary and secondary School [16]. In a population-based study in Sweden, 53% of adolescents aged 14–15 years had uncontrolled asthma [63]. A study in six sub-Saharan African countries reported that 77.7% of adolescents aged 12–14 years attended selected primary and secondary schools with uncontrolled asthma [56], whereas in urban Uganda, 55.5% of school children aged 5–17 years had well-controlled asthma, 29.5% were partly controlled, and 15.0% were poorly controlled [43]. In Brazil, 55.6% of outpatients aged 11 years and older had uncontrolled asthma, with only 17.5% achieving control [12]. These variations highlight differences in study populations, age groups, settings, and healthcare access, reflecting the complexity of asthma management and control globally. The high prevalence of uncontrolled asthma among secondary school-aged adolescents in Kampala may be due to the location of the study, where adolescents are more exposed to environmental triggers.

This study found that environmental determinants, specifically reliance on public transport, were significantly associated with uncontrolled asthma among school going adolescents in Kampala, highlighting the critical role of environmental interventions in asthma management. The school going adolescents with asthma in Kampala who rely on public transport are approximately six times more likely (AOR = 5.75, 95% CI: 1.38, 24.05; *p* = 0.016) to suffer from uncontrolled asthma as compared to those who do not, after adjusting for other variables. The findings are in agreement with that of Mphahlele et al. [44] study done in South Africa where exposure to traffic pollution was associated with severe asthma among school going adolescents. Similar findings were reported in a study that was performed in Southern California [38], where increasing commuting time to school was associated with severe wheezing among children with asthma. Residential traffic-related exposure has been associated with increased asthma severity [71], and on-road and residential exposure have been associated with other acute outcomes [71]. The effects could be due to pollutant exposures that depend on the traffic volume on the roadways traveled, vehicle emission determinants and meteorological conditions. Vehicle emissions are a recognized source of toxic pollutants [36]. A recent study conducted in Kampala by Borràs-Santos et al. [52] found that PM_2.5_ air concentrations in the city surpassed the WHO-recommended limits, even during periods of no vehicle traffic.

This study also revealed that exposure to household moulds (AOR = 3.84, 95% CI: 1.02, 14.46; *p* = 0.047) significantly increased the risk of uncontrolled asthma. Children with moulds in their homes were approximately four times more likely to suffer from uncontrolled asthma as compared to those who do not. Moulds are known asthma triggers, releasing spores and allergens that can exacerbate respiratory symptoms and lead to poor asthma management. Similar findings were reported in other studies, including studies by Borràs-Santos et al. [10] and Alves et al. [4]. However, findings in a study among schoolchildren in urban Uganda [43] differed in that increasing age, concurrent allergic rhinitis and city residence in early life were associated with uncontrolled asthma among children in urban Uganda. This could have been due to the differences in the populations studied, as she looked at children aged 5–17 years.

The strength of this study is that, it is the first to concentrate on adolescents with asthma in secondary schools aged 12–19 years in Kampala, Uganda, since other studies [40, 43, 56] on adolescents with asthma concentrated on different age groups. This study has several limitations. Its cross-sectional design prevents causal inferences, and consecutive sampling of participants introduces the possibility of selection bias. To reduce on the selection bias, we used multistage sampling with probability sampling techniques at primary and secondary stages. We used consecutive sampling to recruit participants to overcome challenges mentioned by Namutebi et al. [46] on recruitment of schoolchildren in studies in Uganda. Participant recruitment relied on school nurse records, potentially excluding children with asthma who lacked a prior asthma record. For example, although a recent study by Oyenuga et al. found severe asthma symptoms among school-going adolescents without a clinician's diagnosis of asthma [56], such individuals were not included in this study, which may have further contributed to selection bias. Recall bias may affect data collected through questionnaires requiring memory of past events and estimations. However, the outcome variable questions were tailored to recall of events within the previous one year. The study did not include lung assessments, such as spirometry, due to limited equipment, trained personnel, and the complexity of interpretation, especially in pediatric populations and in schools [8, 14]. Additionally, concerns about COVID-19 hindered the use of spirometry, as infection-control protocols discouraged aerosol-generating procedures in schools due to the risk of viral transmission [6]. However, the study used standard tools to increase the internal validity. The limited sample size and recruitment from Kampala restrict the generalizability of findings to other regions in Uganda. This study did not measure the air quality in schools however recent evidence indicates that ambient air pollution and weather have an impact on respiratory diseases in Kampala [54]. The study was underpowered to fully assess determinants of uncontrolled asthma. In addition, mixed-effects logistic regression was employed although the prevalence of uncontrolled asthma exceeded 10%; mixed-effects Poisson regression with robust variance could have yielded more appropriate estimates of relative risk. Despite these limitations, the study provides valuable insights into asthma control and its determinants among adolescents in Ugandan secondary schools, highlighting the need for targeted interventions.

## Conclusion

The study reports on the burden of uncontrolled asthma among secondary school adolescents in Kampala. Environmental factors, including reliance on public transportation and exposure to household moulds, were identified as key contributors to poor asthma control, emphasizing the need for targeted interventions such as improved indoor air quality and safer commuting options. We recommend regular asthma screening programs to identify undiagnosed or poorly controlled cases, teacher training to recognize and respond to asthma symptoms, and improved access to inhalers and essential medications within schools. These measures can enhance early detection, timely intervention, and overall asthma management among adolescents in school settings. Although consistent with findings from some global studies, these results differ from others due to variations in study populations, methodologies, and environmental contexts. The strengths of this study include its focus on adolescents and its contribution to understanding asthma control in this demographic. However, limitations such as potential selection and recall biases, reliance on self-reported data, and restricted generalizability to other Ugandan regions call for cautious interpretation. These findings emphasize the urgent need for tailored strategies to improve asthma management among adolescents in Uganda.

## Data Availability

The statistical codes and dataset are available from the OSF repository, [https://osf.io/3qdr6/?view_only = ba3976b659194954af83b959c2a97145](https:/osf.io/3qdr6/?view_only = ba3976b659194954af83b959c2a97145).
